# The effect on vital signs of concomitant administration of nicardipine and dexmedetomidine sedation after spinal anesthesia: A double-blind, randomized controlled trial

**DOI:** 10.1097/MD.0000000000034272

**Published:** 2023-07-07

**Authors:** Sangho Lee, Ye Na Ahn, Junbum Lee, SoonOh Kwon, Hee Yong Kang

**Affiliations:** a Department of Anesthesiology and Pain Medicine, Kyung Hee University College of Medicine, Kyung Hee University Hospital, Seoul, Republic of Korea.

**Keywords:** blood pressure, dexmedetomidine, heart rate, nicardipine, sedation, spinal anesthesia

## Abstract

We evaluated heart rate (HR) and blood pressure (BP) trends when nicardipine (NCD) was co-administered during dexmedetomidine (DEX) sedation after spinal anesthesia.

**Methods:**

Sixty patients aged 19 to 65 were randomly assigned to the DEX or DEX-NCD groups. Five minutes after infusion of the loading dose of DEX, the NCD was administered intravenously at a rate of 5 μg/kg for 5 minutes in the DEX-NCD group. The study starting point was set at 0 minute when the DEX loading dose was initiated. The primary outcomes were the differences in HR and BP between the 2 groups during the study drug administration. Secondary outcomes included the number of patients whose HR was < 50 beats per minute (bpm) after the DEX loading dose infusion, and associated factors were evaluated. The incidence of hypotension in the postanesthesia care unit, postanesthesia care unit length of stay, postoperative nausea and vomiting, postoperative urinary retention, time to first urination after spinal anesthesia, acute kidney injury, and postoperative hospital length of stay were evaluated.

**Results:**

The HR was significantly higher at 14 minutes, and the mean BP was significantly lower at 10 minutes in the DEX-NCD group than in the DEX group. The number of patients with an HR < 50 bpm during surgery was significantly higher in the DEX group than in the DEX-NCD group at 12, 16, 24, 26, and 30 minutes. The DEX group and a low initial HR were independently associated with the occurrence of an HR < 50 bpm after DEX loading dose infusion. Postoperative outcomes were not significantly different between the 2 groups.

**Conclusions:**

Simultaneous administration of NCD during the administration of a loading dose of DEX prevented severe bradycardia. Co-administration of NCD may be considered in patients with a low initial HR when severe bradycardia is expected during the DEX loading dose infusion. NCD and DEX may be safely infused simultaneously without affecting postoperative complications (see Figure S1, Supplemental Digital Content, http://links.lww.com/MD/J241, Graphical abstract).

## 1. Introduction

Spinal anesthesia (SA) is often followed by sedation to ensure patient comfort. Previously, midazolam or propofol have been used to achieve sedation. However, high doses of these agents are associated with potential adverse effects, such as respiratory depression and unstable vital signs, and close monitoring is required.^[1,2]^ Recently, alternative agents such as dexmedetomidine (DEX) have gained popularity as safer and more effective options for sedation following SA.

DEX is a highly selective alpha 2-agonist with physiological effects similar to general sleep.^[3–5]^ Previous studies have been conducted on its use in treating insomnia, delirium, and postoperative cognitive dysfunction.^[6–9]^ In addition, DEX does not suppress the patient’s respiratory function and is a desirable option for sedation compared to other traditional agents.^[10,11]^ DEX is administered as a loading dose followed by a maintenance dose. However, side effects, such as bradycardia, increased blood pressure (BP), dry mouth, and nausea, have been reported during the administration of loading doses.^[12,13]^ High BP and bradycardia during the loading dose may be relieved with the maintenance infusion, and no special treatment is required. However, suddenly elevated or persistently high BP can cause complications such as delayed wound healing or reduced myocardial oxygen supply.^[14]^ Moreover, severe bradycardia may induce cardiac arrest.^[15]^

Nicardipine (NCD) is a well-known calcium channel blocker used to prevent and treat hypertension that occurs during surgery.^[16]^ NCD acts on the smooth muscle of blood vessels as a vasodilator and reduces the preload due to a relative decrease in the effective circulating blood volume, leading to decreased BP.^[17,18]^ Additionally, weak reflexive tachycardia may occur because of a reduction in the preload.^[19]^

Therefore, we hypothesized that simultaneous infusion of NCD during DEX loading administration would prevent hypertension and bradycardia induced by DEX loading dose infusion. This study aimed to evaluate hemodynamic changes during the concomitant administration of these drugs.

## 2. Methods and materials

### 2.1. Study design and ethics

This study was designed to evaluate hemodynamic changes following the concomitant administration of NCD and DEX for sedation after SA in a double-blind, randomized, controlled trial. Ethical approval was obtained from the Institutional Review Board of Kyung Hee University Hospital (KHUH 2017-12-094) on March 23, 2018. The trial was conducted in accordance with the Declaration of Helsinki and registered with the Clinical Research Information Service (No. KCT0002864; registration date, May 15, 2018; principal investigator, Hee Yong Kang). The study protocol was available from the Clinical Research Information Service. Written informed consent was obtained from all participants. The study complied with the Consolidated Standards of Reporting Trials checklist.

### 2.2. Participants

Adult patients aged 19 to 65 who were scheduled for elective lower extremity surgery with sedation under SA at a single tertiary medical center were eligible for trial inclusion. The exclusion criteria were patients who did not require sedation; a SA sensory block level higher than T6; were taking medication for hypertension; had a body mass index ≥ 30 kg/m^2^; had a diagnosis of obstructive sleep apnea; failed SA; were allergic to any study drug; had a preoperative bradycardia with a heart rate (HR) < 60 beats per minute (bpm); had an anesthesia time < 30 minutes; were American Society of Anesthesiologists class ≥ III; had an estimated blood loss > 500 mL; were expected to have a blood transfusion; and/or were pregnant. Recruitment began in March 2018 and ended in December 2018.

### 2.3. Outcomes

The primary outcomes were the differences in HR and BP between the 2 groups during the study drug administration. Vital signs were measured at baseline after entering the operating room, every 2 minutes after drug administration for 30 minutes, and in the postanesthesia care unit (PACU).

The secondary outcomes were the number of patients whose HR was < 50 bpm after the DEX loading dose infusion and the associated factors. The incidence of hypotension in the PACU, PACU length of stay (LOS), postoperative nausea and vomiting (PONV), postoperative urinary retention (POUR), time to first urination after SA, acute kidney injury (AKI), and postoperative hospital LOS were evaluated. Hypotension in the PACU was defined as a decrease in systolic BP to <20% of the baseline. PONV was defined as a complaint of nausea or vomiting for which antiemetic drugs were administered. POUR was defined as urine retention of ≥300 mL on clean intermittent catheterization while the patient complained of suprapubic discomfort.^[20,21]^ AKI was diagnosed using the Kidney Disease: Improving Global Outcomes criteria and was defined as an alteration of the serum creatinine level ≥ 0.3 mg/dL until postoperative day (POD) 2.^[22]^

### 2.4. Randomization and masking

The patients were randomly assigned to the intervention (DEX and NCD, DEX-NCD) or control (DEX and normal saline, DEX) groups using sealed opaque envelopes on the morning of surgery. A computer-generated random allocation sequence was created using Excel 2019 (Microsoft) with a 1:1 allocation and random block sizes. For double-blindness, 1 researcher (S.K.), who did not manipulate the study data, confirmed the group assignment and administered the NCD or saline at a fixed infusion rate.

### 2.5. Procedures

After entering the operating room, HR and rhythm were monitored using a 3-lead electrocardiogram, noninvasive BP was monitored using an arm cuff, and peripheral oxygen saturation was recorded for all participants. Vital signs were recorded every 2 minutes for 30 minutes after the SA. Prior to SA, 5 mL/kg crystalloid was rapidly administered to prevent hypotension due to peripheral vasodilation. For the SA, hyperbaric bupivacaine 0.5% was injected intrathecally using a 25G spinal needle with an interlaminar approach in the lateral position. The dose of bupivacaine was within the range of 11 to 14 mg, according to the patient’s height and expected operation time. The patient was placed supine, and the complete loss of cold or light touch sensation determined the level of blockage. Once the blockage level was fixed, DEX (1 μg/kg) was administered intravenously (IV) to both groups at the loading dose for 10 minutes. The starting time point for the study was set at 0 minute when the DEX loading dose was initiated after SA. After 5 minutes of loading dose administration, 1 researcher (S.K.) confirmed the group assignment and the dose of NCD (60 μg/mL) at 5 μg/kg was administered within 5 minutes in the DEX-NCD group. In the DEX (control) group, an equal volume of normal saline was infused IV as a placebo. After infusion of the loading dose for 10 minutes, the DEX dose was changed to a maintenance dose of 0.5 μg/kg/h. The infusion device used in both groups was the TERUFUSION^®^ INFUSION PUMP TE-171 (Terumo^®^, Tokyo, Japan). The 2 study drugs were administered simultaneously via an IV line. The infusion line was connected to the most proximal part of the patient’s IV catheter to allow immediate drug infusion.

Intraoperative hypotension and hypertension were defined as changes in systolic BP of >20% from baseline. In the case of hypotension, we planned to administer a vasoconstrictor or inotropic agent, depending on the situation; in the case of hypertension, NCD or a beta-blocker was administered. At the end of the surgery, fentanyl-based (0.35 μg/kg/h) IV patient-controlled analgesia was administered to both groups until POD 2.

### 2.6. Outcome assessments and data collection

During the perioperative period, the patient was monitored, and vital signs were recorded from admission to the operating room to the PACU.

Based on medical records, PONV, POUR, time to first urination after SA, and AKI were evaluated until POD 2. The medical staff documented the first urination time in the medical records through patient self-reporting. Additionally, the incidence of hypotension in the PACU, PACU LOS, and postoperative hospital LOS were evaluated.

### 2.7. Sample size calculation

A pilot study was conducted at our hospital for SA patients using the same procedure used in this study. In the pilot study, the HR at 10 minutes after DEX infusion was 64.5 ± 6.9 bpm versus 57.1 ± 6.2 bpm in the DEX-NCD and DEX groups, respectively. Using G-power analysis with these data (*t* test, means: difference between 2 independent means (2 groups); A priori: Compute required sample size—2 tails; Effect size *d*, 1.13; α err, 0.05; power, 0.95; allocation ratio N2/N1, 1), a sample size of 22 participants in each group was calculated. Considering a 25% dropout rate, the target number of participants was 60, with 30 patients in each group.

### 2.8. Statistical analysis

Data are presented as the median (interquartile range) or number (%). Categorical variables were analyzed using Fisher’s exact test or the chi-squared test. The normality of continuous variables was evaluated using the Shapiro–Wilk test. Independent variable t-tests or Wilcoxon rank-sum tests were used to analyze continuous variables. Univariate logistic regression analysis was used to explore the factors causing the HR to be < 50 bpm; variables with a *P* value < .20 and previously described clinically important factors were included in the multivariate logistic regression analysis. Statistical significance was set at a *P* value < .05. Statistical analyses were performed using SPSS (version 22.0; SPSS Inc., Chicago, IL).

## 3. Results

### 3.1. Study population, demographic, and intraoperative data

**Figure 1.**
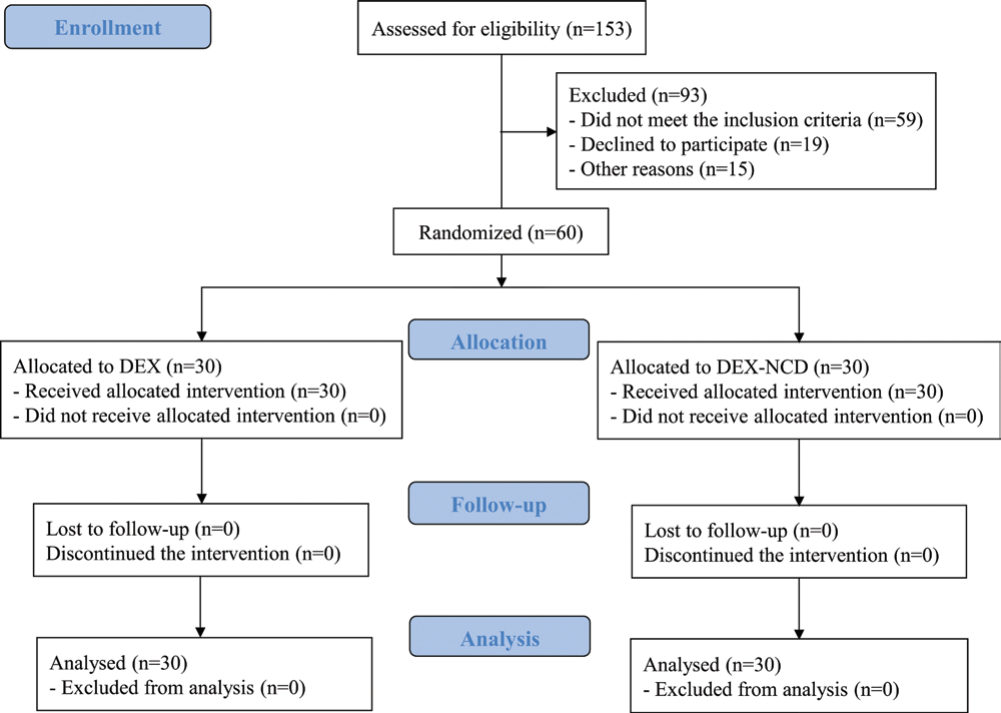
CONSORT diagram of the patient flowchart. CONSORT = Consolidated Standards of Reporting Trials, DEX = dexmedetomidine, DEX-NCD = dexmedetomidine-nicardipine.

A total of 153 patients met the eligibility criteria; 93 were excluded, and 60 were randomly assigned. Thirty participants were equally assigned to both groups without loss to follow-up. Finally, 30 patients in each group were analyzed (Fig. 1). The demographic data, preoperative laboratory findings, bupivacaine dose, sensory block level, surgical time, the incidence of intraoperative hypertension and hypotension, and type of surgery did not differ significantly between the 2 groups (Table 1). None of the study participants developed a serious arrhythmia, other than bradycardia, during the surgery.

**Table 1:** Patient characteristics and intraoperative data of the study cohort.

	DEX (n = 30)	DEX-NCD (n = 30)	*P* value
Demographics			
Age (yr)	40 (27–56)	39 (28–53)	.773
Sex (male/female)	18/12	15/15	.604
BMI (kg/m^2^)	25.6 (22.6–26.9)	23.9 (21.5–26.6)	.201
ASA class (I/II)	18/12	23/7	.267
White blood cell (×10^3^/μL)	5.73 (4.81–7.27)	6.35 (5.54–7.34)	.171
Hemoglobin (g/dL)	14.2 (13.1–15.2)	14.3 (13.5–15.5)	.569
Platelet (×10^3^/μL)	239 (205–283)	247 (222–291)	.487
AST (U/L)	23 (20–26)	22 (19–24)	.306
ALT (U/L)	23 (16–31)	18 (14–28)	.231
Creatinine (mg/dL)	0.8 (0.6–0.9)	0.7 (0.6–0.8)	.451
Intraoperative data			
Dose of bupivacaine (mg)	13 (12–13)	12 (12–13)	.446
Sensory block level (n)			
T6	5 (16.7%)	3 (10.0%)	.714
T8	11 (36.7%)	10 (33.3%)	
T10	11 (36.7%)	15 (50.0%)	
T12	3 (10.0%)	2 (6.7%)	
Surgical time (min)	55 (40–65)	65 (30–80)	.841
Intraoperative hypertension (n)	5 (16.7%)	3 (10.0%)	.704
Intraoperative hypotension (n)	2 (6.7%)	3 (10.0%)	1.000
Type of surgery (n)			
Knee arthroscopy	18 (60.0%)	14 (46.7%)	.913
Fixative removal	5 (16.7%)	7 (23.3%)	
Mass excision	3 (10.0%)	3 (10.0%)	
High tibia osteotomy	2 (6.7%)	2 (6.7%)	
ORIF	1 (3.3%)	2 (6.7%)	
Other	1 (3.3%)	2 (6.7%)	

Data are presented as medians (interquartile ranges) or numbers (%).

ALT = alanine transaminase, ASA = American Society of Anesthesiologists, AST = aspartate transaminase, BMI = body mass index, DEX = dexmedetomidine, DEX-NCD = dexmedetomidine-nicardipine, ORIF = Open Reduction and Internal Fixation, T = thoracic.

### 3.2. Primary outcome

**Figure 2.**
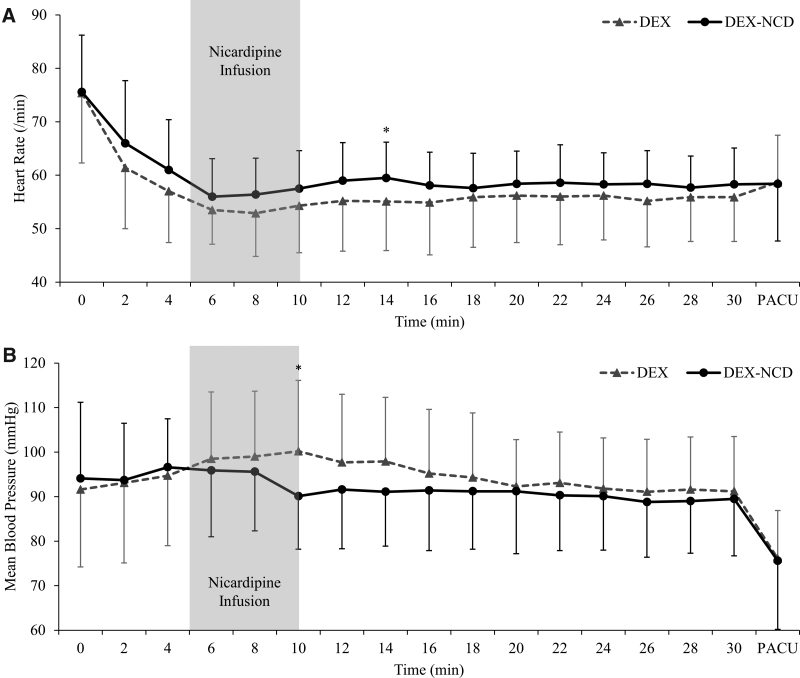
Heart rate and mean blood pressure trends at different time points after dexmedetomidine administration. The gray zone is the period of nicardipine infusion in the DEX-NCD group. (A) Heart rate. (B) Mean blood pressure. Data are presented as means ± standard deviations. *Significant differences between the 2 groups at each time point. DEX = dexmedetomidine, DEX-NCD = dexmedetomidine-nicardipine, PACU = postanesthesia care unit.

Overall, the intraoperative HR tended to remain higher in the DEX-NCD group than in the DEX group. In particular, the HR was significantly higher at 14 minutes in the DEX-NCD group (Fig. 2A; Table S1, Supplemental Digital Content, http://links.lww.com/MD/J242). The mean BP (mBP) was significantly higher in the DEX group than in the DEX-NCD group at 10 minutes (Fig. 2B; Table S2, Supplemental Digital Content, http://links.lww.com/MD/J243). However, the 2 groups showed no significant differences in HR and mBP at the remaining time points.

### 3.3. Secondary outcomes

**Figure 3.**
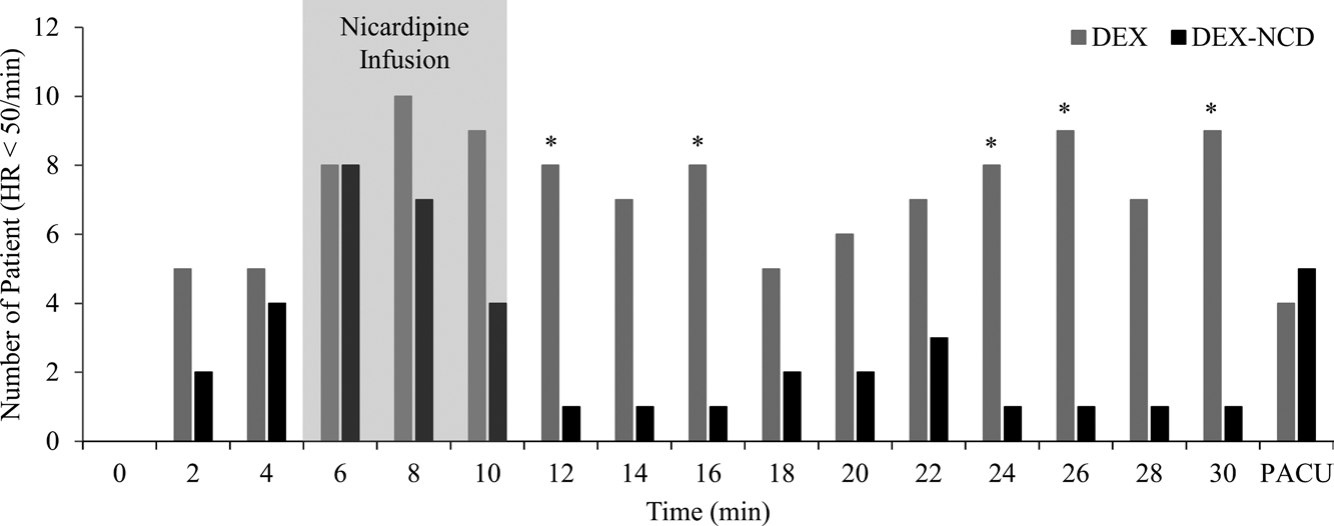
The number of patients with HR < 50 beats per min during the perioperative period. The gray zone is the period of nicardipine infusion in the DEX-NCD group. *Significant differences between the 2 groups at each time point. DEX = dexmedetomidine, DEX-NCD = dexmedetomidine-nicardipine, HR = heart rate, PACU = postanesthesia care unit.

**Figure 4.**
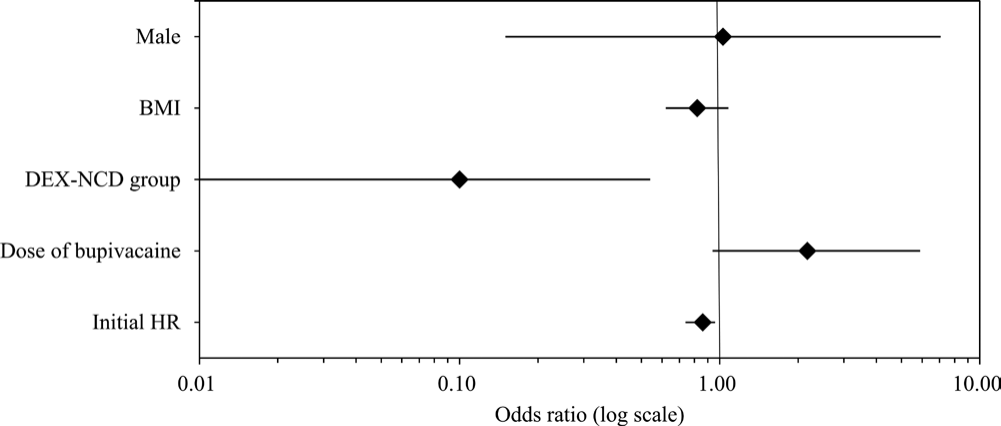
Forest plot of the odds ratios for an HR < 50 beats per min after nicardipine infusion. BMI = body mass index, DEX-NCD = dexmedetomidine-nicardipine, HR = heart rate.

The number of patients with an HR < 50 bpm after the DEX loading dose infusion was significantly higher in the DEX group than in the DEX-NCD group at 12, 16, 24, 26, and 30 minutes after DEX administration (Fig. 3; Table S3, Supplemental Digital Content, http://links.lww.com/MD/J244). Univariate regression analysis of the perioperative factors that caused an HR of <50 bpm showed that the DEX-NCD group, bupivacaine dose, and initial HR were significantly associated with an HR of <50 bpm (Table 2). Multivariate regression analysis that included all significant variables derived from the univariate analysis, as well as clinically meaningful variables, showed that the DEX-NCD group and the initial HR were significantly associated with an HR < 50 bpm after the DEX loading dose infusion (Table 2; Fig. 4).

**Table 2:** Univariable and multivariable logistic regression analysis of factors associated with an HR < 50 beats per min after nicardipine infusion.

	Univariable		Multivariable	
	OR (95% CI)	*P* value	OR (95% CI)	*P* value
Age	1.00 (0.95–1.04)	.834		
Male	0.40 (0.10–1.39)	.166	1.03 (0.15–7.07)	.978
BMI (kg/m^2^)	0.95 (0.79–1.15)	.592	0.82 (0.62–1.08)	.152
ASA	1.27 (0.34–4.40)	.710		
DEX-NCD group	0.19 (0.04–0.71)	.021*	0.10 (0.01–0.54)	.015*
Bupivacaine (mg)	2.30 (1.26–4.65)	.011*	2.17 (0.94–5.90)	.089
Block level	1.00 (0.70–1.46)	.985		
Initial mBP (mm Hg)	0.99 (0.96–1.03)	.745		
Initial HR (/min)	0.84 (0.73–0.93)	.005*	0.86 (0.74–0.96)	.022*

ASA = American Society of Anesthesiologists, BMI = body mass index, CI = confidence interval, DEX-NCD = dexmedetomidine-nicardipine, HR = heart rate, mBP = mean blood pressure, OR = odds ratio.

* Statistical significance.

Postoperative outcomes such as hypotension and LOS in the PACU were not significantly different between the 2 groups. PONV, POUR, time to first urination after SA, and postoperative creatinine levels were also comparable between the groups. AKI did not occur in either group until POD 2, and there was no significant difference in the postoperative hospital LOS between the 2 groups (Table 3).

**Table 3:** Postoperative data of the study cohort.

	DEX (n = 30)	DEX-NCD (n = 30)	*P* value
Hypotension in PACU	12 (40.0%)	13 (43.3%)	1.000
PACU LOS (min)	50 (32.0–67.0)	50 (37.0–62.0)	.994
PONV until POD2	7 (23.3%)	8 (26.7%)	1.000
Postoperative urinary retention	3 (10.0%)	2 (6.7%)	1.000
Time to first urination after SA (min)	467 (361–560)	430 (354–515)	.425
Postoperative creatinine (mg/dL)	0.8 (0.6–0.8)	0.7 (0.6–0.8)	.544
AKI until POD2	0 (0%)	0 (0%)	NA
Postoperative hospital LOS (d)	3 (1–4)	3 (2–4)	.862

Data are presented as medians (interquartile ranges) or numbers (%).

AKI = acute kidney injury, DEX = dexmedetomidine, DEX-NCD = dexmedetomidine-nicardipine, LOS = length of stay, PACU = postanesthesia care unit, POD = postoperative days, PONV = postoperative nausea and vomiting, SA = spinal anesthesia.

## 4. Discussion

In patients undergoing lower extremity surgery under SA, concomitant administration of NCD during the DEX loading dose infusion increased the intraoperative HR and decreased the mBP. The administration of NCD also reduced the occurrence of severe bradycardia (HR < 50 bpm). An intraoperative HR of <50 bpm was associated with a lower initial HR and DEX administration without NCD. Kim and Ahn^[23]^ also reported that an initial low HR was a risk factor for DEX-induced bradycardia. This result was comparable to the current study’s, and caution should be exercised when using DEX in patients with a low initial HR.

The cardiovascular effects of DEX begin with initial hypertension following the administration of the loading dose due to the activation of α2B receptors located on vascular smooth muscle, with subsequent hypotension and bradycardia due to a centrally mediated decrease in the sympathetic tone.^[24,25]^ During the loading dose infusion, DEX mainly acts on the α2B receptors, and NCD also acts on the α2 receptor. Therefore, NCD, rather than other drugs, was selected for this study. Kang et al^[26]^ administered atropine and ephedrine to treat DEX-induced bradycardia. Although the bradycardia resolved with these drugs, the BP increased further. The administration of NCD may be more effective in maintaining a stable HR and BP. Therefore, when DEX is administered to a patient with a low initial HR, severe bradycardia may be prevented by the simultaneous administration of NCD. Postoperative data were comparable between the 2 groups, and the co-administration of DEX and NCD may be safely considered.

Since the rapid initial distribution half-life of NCD is 2.7 minutes,^[16]^ the 2 groups showed no differences in postoperative outcomes. The NCD was administered 5 minutes after the start of the DEX loading because, in the pilot study we conducted, the HR decreased by ≥20% compared to baseline at 5 minutes of DEX loading. In addition, NCD administration was terminated at the end of DEX loading because of the relatively short half-life of NCD. The HR and BP were significantly different only at time points close to the time of NCD administration, with no significant difference between the 2 groups after 14 minutes. However, the trends of high HR and low BP were maintained for 30 minutes in the DEX-NCD group. In addition, the number of patients whose HR decreased to <50 bpm differed significantly between the 2 groups, even after 30 minutes. This may be because the HR during NCD administration was maintained at a constant level even after NCD administration was stopped.

Shan et al^[27]^ and Soh et al^[28]^ reported that DEX effectively prevents AKI because of its sympatholytic, antioxidative, and anti-inflammatory effects.^[29,30]^ According to studies by Kim et al^[31]^ and Simonin et al,^[32]^ the incidences of AKI after SA were 3.0% and 5.1%, respectively. Although there were differences in patient age and medical histories compared with those in previous studies, AKI did not occur in the current study, and creatinine levels did not significantly change before and after surgery, which may be related to the reno-protective effect of DEX.

Additionally, DEX has been reported to increase urine output.^[33–35]^ In this study, POUR occurred in 5 patients (8.3%). Niazi and Taha^[36]^ reported that the incidence of POUR was approximately 20% when midazolam was administered as a sedative after SA. Haleem et al^[37]^ reported that the incidence of POUR after SA without sedation is 10%. However, both studies defined POUR as 500 ml of residual urine. If the same criteria were applied to the current study, the incidence of POUR would decrease further. The low incidence of POUR may be due to increased urine output during DEX administration. Further research should be conducted on the relationships among DEX use, urine output, and POUR.

### 4.1. Limitations

This study had some limitations. First, the study was conducted on relatively young and healthy patients who underwent numerous types of lower extremity surgeries. However, since resting HR tends to be slow in young and healthy populations,^[38,39]^ the strength of this study was the co-administration of an NCD, which prevented DEX-induced bradycardia. In addition, when only lower extremity surgery was included, the surgical position, method of anesthesia, and hemodynamic changes were relatively consistent. There was no significant difference in the type of surgery performed between the 2 groups.

Second, this study included a relatively small number of participants. We sought to control confounding factors by recruiting patients using strict exclusion criteria. However, there were no differences in the demographic data and postoperative outcomes between the 2 groups; therefore, randomization was effective. In addition, significant differences were observed in vital signs during the study drug administration, and the number of participants was not unreasonably small.

Third, vital signs were observed for only 30 minutes during the study drug administration. However, considering the duration of the action of NCD, evaluating vital signs until 20 minutes after NCD administration may be reasonable.^[40]^

## 5. Conclusions

The simultaneous administration of the NCD prevented severe bradycardia during DEX loading dose infusion. In particular, for patients with a low initial HR, the co-administration of NCD may be considered when severe bradycardia is expected during DEX loading dose infusion. Concomitant administration of NCD during DEX infusion did not affect postoperative complications. Further large-scale trials should be conducted with patients in an intensive care setting, with older adults, and those undergoing upper extremity surgery.

## Acknowledgments

Statistical analyses were conducted after consulting Jae-Hong Ryoo (Kyung Hee University College of Medicine), a statistical expert.

## Author contributions

**Conceptualization:** Sangho Lee, Ye Na Ahn, Junbum Lee, SoonOh Kwon, Hee Yong Kang.

**Data curation:** Sangho Lee, Ye Na Ahn.

**Formal analysis:** Hee Yong Kang.

**Investigation:** Sangho Lee, Junbum Lee.

**Methodology:** Ye Na Ahn, Hee Yong Kang.

**Project administration:** Hee Yong Kang.

**Resources:** SoonOh Kwon.

**Software:** Sangho Lee, Hee Yong Kang.

**Supervision:** Hee Yong Kang.

**Visualization:** Sangho Lee, Junbum Lee.

**Writing – original draft:** Sangho Lee.

**Writing – review & editing:** Hee Yong Kang.

## Supplementary Material








