# Supplementary figures and images for: The effect on vital signs of concomitant administration of nicardipine and dexmedetomidine sedation after spinal anesthesia: A double-blind, randomized controlled trial
Source: Medicine (Baltimore). 2023 Jul 7;102(27):e34272. doi: 10.1097/MD.0000000000034272 (PMC10328642; doi:10.1097/MD.0000000000034272)

Supplementary Figure S1. Graphical abstract

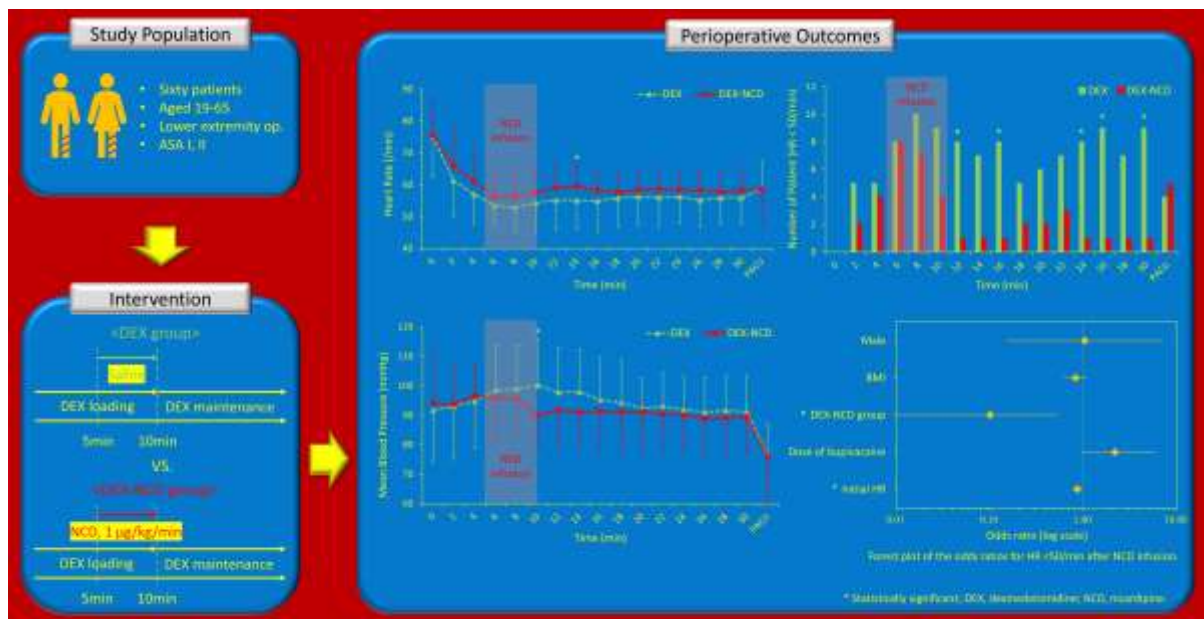

Supplement: Supplementary file 1 [file medi-102-e34272-s001.pdf]
