# Supplementary material for: The effect on vital signs of concomitant administration of nicardipine and dexmedetomidine sedation after spinal anesthesia: A double-blind, randomized controlled trial
Source: Medicine (Baltimore). 2023 Jul 7;102(27):e34272. doi: 10.1097/MD.0000000000034272 (PMC10328642; doi:10.1097/MD.0000000000034272)
Supplement: Supplementary file 2 [file medi-102-e34272-s002.pdf]

Table S1. Perioperative heart rate at different stages.

	DEX	DEX-NCD	*P* value
0 min	75.4 ± 13.1	75.6 ± 10.6	.948
2 min	61.4 ± 11.4	66.0 ± 11.7	.128
4 min	57.0 ± 9.6	61.0 ± 9.4	.113
6 min	53.5 ± 6.4	56.0 ± 7.1	.161
8 min	52.9 ± 8.1	56.4 ± 6.8	.073
10 min	54.3 ± 8.8	57.5 ± 7.1	.134
12 min	55.2 ± 9.4	59.0 ± 7.1	.076
14 min	55.1 ± 9.2	59.5 ± 6.7	.038*
16 min	54.9 ± 9.8	58.1 ± 6.2	.145
18 min	55.9 ± 9.4	57.6 ± 6.5	.409
20 min	56.2 ± 8.8	58.4 ± 6.1	.257
22 min	56.0 ± 9.0	58.6 ± 7.1	.214
24 min	56.2 ± 8.3	58.3 ± 5.9	.279
26 min	55.2 ± 8.6	58.4 ± 6.2	.110
28 min	55.9 ± 8.3	57.7 ± 5.9	.329
30 min	55.9 ± 8.3	58.3 ± 6.8	.233
PACU	58.7 ± 8.8	58.4 ± 10.7	.906

Data are presented as means ± standard deviations. *Statistical significance. DEX, dexmedetomidine; DEX-NCD, dexmedetomidine-nicardipine; PACU, postanesthesia care unit.
