# Supplementary material for: The effect on vital signs of concomitant administration of nicardipine and dexmedetomidine sedation after spinal anesthesia: A double-blind, randomized controlled trial
Source: Medicine (Baltimore). 2023 Jul 7;102(27):e34272. doi: 10.1097/MD.0000000000034272 (PMC10328642; doi:10.1097/MD.0000000000034272)
Supplement: Supplementary file 3 [file medi-102-e34272-s003.pdf]

Table S2. Perioperative mean blood pressure at different stages.

	DEX	DEX-NCD	*P* value
0 min	91.6 ± 17.4	94.1 ± 17.1	.572
2 min	93.1 ± 18.0	93.7 ± 12.8	.876
4 min	94.7 ± 15.7	96.6 ± 10.9	.575
6 min	98.5 ± 15.0	95.9 ± 14.9	.498
8 min	99.0 ± 14.7	95.6 ± 13.3	.352
10 min	100.2 ± 15.9	90.1 ± 11.9	.007*
12 min	97.7 ± 15.3	91.6 ± 13.3	.102
14 min	97.9 ± 14.4	91.1 ± 12.2	.055
16 min	95.2 ± 14.4	91.4 ± 13.5	.304
18 min	94.3 ± 14.5	91.2 ± 13.0	.382
20 min	92.3 ± 10.5	91.2 ± 14.0	.717
22 min	93.1 ± 11.4	90.3 ± 12.4	.354
24 min	91.8 ± 11.4	90.1 ± 12.1	.577
26 min	91.1 ± 11.8	88.8 ± 12.4	.457
28 min	91.6 ± 11.8	89.0 ± 11.7	.382
30 min	91.2 ± 12.3	89.5 ± 12.8	.610
PACU	76.1 ± 10.8	75.6 ± 15.4	.885

Data are presented as means ± standard deviations. *Statistical significance. DEX, dexmedetomidine; DEX-NCD, dexmedetomidine-nicardipine; PACU, postanesthesia care unit.
