# Supplementary material for: The effect on vital signs of concomitant administration of nicardipine and dexmedetomidine sedation after spinal anesthesia: A double-blind, randomized controlled trial
Source: Medicine (Baltimore). 2023 Jul 7;102(27):e34272. doi: 10.1097/MD.0000000000034272 (PMC10328642; doi:10.1097/MD.0000000000034272)
Supplement: Supplementary file 4 [file medi-102-e34272-s004.pdf]

Table S3. The number of patients whose perioperative heart rate was <50 beats per min.

	DEX	DEX-NCD	*P* value
0 min	0 (0.0%)	0 (0.0%)	NA
2 min	5 (16.7%)	2 (6.7%)	.421
4 min	5 (16.7%)	4 (13.3%)	1.000
6 min	8 (26.7%)	8 (26.7%)	1.000
8 min	10 (33.3%)	7 (23.3%)	.567
10 min	9 (30.0%)	4 (13.3%)	.210
12 min	8 (26.7%)	1 (3.3%)	.030*
14 min	7 (23.3%)	1 (3.3%)	.058
16 min	8 (26.7%)	1 (3.3%)	.030*
18 min	5 (16.7%)	2 (6.7%)	.421
20 min	6 (20.0%)	2 (6.7%)	.255
22 min	7 (23.3%)	3 (10.0%)	.299
24 min	8 (26.7%)	1 (3.3%)	.030*
26 min	9 (30.0%)	1 (3.3%)	.015*
28 min	7 (23.3%)	1 (3.3%)	.058
30 min	9 (30.0%)	1 (3.3%)	.015*
PACU	4 (13.3%)	5 (16.7%)	1.000

Data are presented as means ± standard deviations. * Statistical significance. DEX, dexmedetomidine; DEX-NCD, dexmedetomidine-nicardipine; PACU, postanesthesia care unit.
